# A Case of Head Injury, with Spontaneous Trephination

**Published:** 1879-09

**Authors:** R. W. Westmoreland

**Affiliations:** Atlanta, Ga.


### A CASE OF HEAD INJURY, WITH SPONTANEOUS TREPHINATION.

By R. W. WESTMORELAND, M.D., Atlanta, Ga.

Wm. Blank, æt twenty-two, received on the afternoon of the 24th of April, an incised wound of the scalp, on the left side, just above, and posterior to the mastoid process, from a blow with a weeding hoe. The wound was about two inches long, in a vertical direction, and ranged forwards. In connection with the external wound, it was found that the outer table of the cranium was indented, but no loose fragments of bone were discovered. He walked a mile or more, after the reception of the injury, to my office, and presented himself free of any alarming symptoms. After dressing the wound he was directed to go home, keep quiet and let me know if he did not get along well, as I was inclined to anticipate further trouble of a more or less serious character.

I was sent for during the night, and found the patient in a semi-comatose condition with full, slow pulse, but not the complete loss of sensibility and consciousness that is usual in compression of the brain. Concluding that the trouble was from extravasation of blood between the membranes as a result of the blow, and not from depressed bone, it was decided to give an opiate and await results to decide upon the trephine. Accordingly, a quarter of a grain of morphia was given hypodermically, and he was left for the night.

Upon seeing the patient the next morning, he was found to be much better. The symptoms of compression had given place to what more resembled those of concussion, and considerable pain was complained of at the point of injury. On the third day the wound of the scalp which had healed up, was opened and discharged pus constantly. Paraplegia of the right side came on the fifth day, but disappeared under the use of strychnine and bromide of potash in two or three days.

On the third or fourth week, while probing for necrosed bone, the point of the probe entered a small opening of the cranium, and immediately there flowed out a considerable amount of pus. It was thought that there were loose fragments of bone, but none were ever extracted, nor was any ever discovered in the discharges. After the opening of the internal abscess the patient gradually improved, and in about four weeks from the reception of the injury was considered out of danger.

The points that are considered of interest in the above report are the delay in the appearance of symptoms indicating brain injury, the commingling of symptoms of compression and concussion, the promptness with which these mechanical brain lesions were controlled, the formation and course of the meningeal abscess, and finally, the death, discharge and reparation of the injured bone.

As to the first point, it is not altogether rare that, though there is decided shock at the reception of the blow, the patient may recover, and not experience any further trouble for some hours, when the symptoms of brain lesion may assert themselves. In the case reported there was *no* shock at first. He was neither "staggered" nor had he "mental confufusion" from the blow. Upon this fact is based the theory that while the blow was not sufficiently forcible to produce the molecular vibration necessary to a disturbance of the brain function at once, it did produce separation between the dura mater and cranium, followed of course, by extravasation of blood. The simple vibration added to the latter produced the complication of symptoms, and a simple form of compression and concussion of the brain existed at the same time.

The outer plate of the cranium was fractured, but not sufficiently to be considered of much moment at the time, and yet the resulting morbid processes accomplished spontaneously trephination, which, otherwise, might have required instrumental interference.

